# Rapid identification of a human antibody with high prophylactic and therapeutic efficacy in three animal models of SARS-CoV-2 infection

**DOI:** 10.1073/pnas.2010197117

**Published:** 2020-11-02

**Authors:** Wei Li, Chuan Chen, Aleksandra Drelich, David R. Martinez, Lisa E. Gralinski, Zehua Sun, Alexandra Schäfer, Swarali S. Kulkarni, Xianglei Liu, Sarah R. Leist, Doncho V. Zhelev, Liyong Zhang, Ye-Jin Kim, Eric C. Peterson, Alex Conard, John W. Mellors, Chien-Te K. Tseng, Darryl Falzarano, Ralph S. Baric, Dimiter S. Dimitrov

**Affiliations:** ^a^Department of Medicine, Division of Infectious Diseases, Center for Antibody Therapeutics, University of Pittsburgh Medical School, Pittsburgh, PA 15261;; ^b^Department of Microbiology and Immunology, Centers for Biodefense and Emerging Diseases, Galveston National Laboratory, Galveston, TX 77550;; ^c^Department of Epidemiology, University of North Carolina at Chapel Hill, Chapel Hill, NC 27599;; ^d^Department of Veterinary Microbiology, Vaccine and Infectious Disease Organization–International Vaccine Centre, University of Saskatchewan, Saskatoon, SK S7N 5E3, Canada;; ^e^Abound Bio, Pittsburgh, PA 15219

**Keywords:** therapeutic antibodies, coronaviruses, SARS-CoV-2, animal models

## Abstract

Effective therapies are urgently needed for the SARS-CoV-2/COVID-19 pandemic. We identified panels of fully human monoclonal antibodies (mAbs) from large phage-displayed Fab, scFv, and VH libraries by panning against the receptor binding domain (RBD) of the SARS-CoV-2 spike (S) glycoprotein. A high-affinity Fab was selected from one of the libraries and converted to a full-size antibody, IgG1 ab1, which competed with human ACE2 for binding to RBD. It potently neutralized replication-competent SARS-CoV-2 but not SARS-CoV, as measured by two different tissue culture assays, as well as a replication-competent mouse ACE2-adapted SARS-CoV-2 in BALB/c mice and native virus in hACE2-expressing transgenic mice showing activity at the lowest tested dose of 2 mg/kg. IgG1 ab1 also exhibited high prophylactic and therapeutic efficacy in a hamster model of SARS-CoV-2 infection. The mechanism of neutralization is by competition with ACE2 but could involve antibody-dependent cellular cytotoxicity (ADCC) as IgG1 ab1 had ADCC activity in vitro. The ab1 sequence has a relatively low number of somatic mutations, indicating that ab1-like antibodies could be quickly elicited during natural SARS-CoV-2 infection or by RBD-based vaccines. IgG1 ab1 did not aggregate, did not exhibit other developability liabilities, and did not bind to any of the 5,300 human membrane-associated proteins tested. These results suggest that IgG1 ab1 has potential for therapy and prophylaxis of SARS-CoV-2 infections. The rapid identification (within 6 d of availability of antigen for panning) of potent mAbs shows the value of large antibody libraries for response to public health threats from emerging microbes.

## Significance

Effective therapies are urgently needed for COVID-19. We rapidly (within a week) identified a fully human monoclonal germline-like antibody (ab1) from phage-displayed libraries that potently inhibited mouse ACE2-adapted SARS-CoV-2 replication in wild-type BALB/c mice and native virus in transgenic mice expressing human ACE2 as well as in hamsters when administered before virus challenge. It was also effective when administered after virus infection of hamsters, although at lower efficacy than when used prophylactically. Ab1 was highly specific and did not bind to human cell membrane-associated proteins. It also exhibited good developability properties including complete lack of aggregation. Ab1 has potential for prophylaxis and therapy of COVID-19 alone or in combination with other agents.

The severe acute respiratory distress syndrome coronavirus 2 (SARS-CoV-2) (1) has spread worldwide thus requiring safe and effective prevention and therapy. Inactivated serum from convalescent patients inhibited SARS-CoV-2 replication and decreased symptom severity of newly infected patients (2), suggesting that monoclonal antibodies (mAbs) could be even more effective. Human mAbs are typically highly target specific and relatively nontoxic. By using phage display we have previously identified a number of potent fully human mAbs (m396, m336, and m102.4) against emerging viruses, including severe acute respiratory syndrome coronavirus (SARS-CoV) (3), Middle East respiratory syndrome coronavirus (MERS-CoV) (4), and henipaviruses (5, 6), respectively, which are also highly effective in animal models of infection (7–10); one of them was administered on a compassionate basis to humans exposed to henipaviruses and successfully evaluated in a clinical trial (11).

Size and diversity of phage-displayed libraries are critical for rapid selection of high-affinity antibodies without the need for additional affinity maturation. Our exceptionally potent antibody against the MERS-CoV, m336, was directly selected from a very large (size ∼10^11^ clones) library from 50 individuals (4). However, another potent antibody, m102.4, against henipaviruses was additionally affinity matured from its predecessor selected from a smaller library (size ∼10^10^ clones) from 10 individuals (6). Thus, to generate high-affinity and safe mAbs we used very large (size ∼10^11^ clones each) naive human antibody libraries in Fab, scFv, or VH format using peripheral blood mononuclear cells (PBMCs) from a total of 490 individuals obtained before the SARS-CoV-2 outbreak. The complementarity-determining regions (CDRs) of the human VH domains were grafted (except CDR1 which was mutagenized or grafted) from our other libraries as previously described (12).

Another important factor to consider when selecting effective mAbs is the appropriate antigen. Similar to SARS-CoV, SARS-CoV-2 uses the spike (S) glycoprotein to enter into host cells. The S receptor binding domain (RBD) binds to its receptor, the human angiotensin-converting enzyme 2 (hACE2), thus initiating a series of events leading to virus entry into cells (13). We have previously characterized the function of the SARS-CoV S glycoprotein and identified its RBD which is stable in isolation (14). The RBD was then used as an antigen to pan phage-displayed antibody libraries; we identified potent antibodies (4, 7) more rapidly and the antibodies were more potent than when we used the whole S protein or S2 as panning antigens. In addition, the SARS-CoV RBD-based immunogens are highly immunogenic and elicit neutralizing antibodies which protect against SARS-CoV infections (15). Thus, to identify SARS-CoV-2 mAbs, we generated two variants of the SARS-CoV-2 RBD (amino acids [aa] 330 to 532) (*SI Appendix*, Fig. S1) and used them as antigens for panning of our libraries.

## Results and Discussion

### Identification of High-Affinity Human Antibodies in Different Formats Targeting the SARS-CoV-2 RBD.

Panels of high-affinity binders to RBD in Fab, scFv, and VH domain formats were identified from our antibody phage libraries. There was no preferential use of any antibody VH gene (an example for a panel of binders selected from the scFv library is shown in *SI Appendix*, Fig. S2*A*) and the number of somatic mutations was relatively low (*SI Appendix*, Fig. S2*B*, for the same panel of binders as in *SI Appendix*, Fig. S2*A*). The antibodies bound to SARS-CoV-2 RBD with half-maximal effective concentrations ranging from 1 to 1,000 nM (*SI Appendix*, Fig. S3*A*). The highest-affinity binders were converted to the IgG1 and VH-Fc fusion formats to increase binding through avidity and half-life in vivo. Some of them including ab1, 2, 3, 9, and m398 competed to various degrees with hACE2, while others including ab5, m399, m400, and m401 did not (*SI Appendix*, Fig. S3*B*). The hACE2-competing antibodies ab2, 3, 9, and m398 competed with ab1, while the hACE2-noncompeting antibodies did not compete with ab1 for binding to RBD (*SI Appendix*, Fig. S3*C*). The m398 which competed with hACE2 relatively weakly also competed weakly with CR3022, indicating that it has a distinct epitope compared to the epitopes of the antibodies (ab1, 2, and 3) which competed strongly with hACE2 (*SI Appendix*, Fig. S3*D*). None of the antibodies cross-reacted with the SARS-CoV S1 except ab5 which exhibited weak cross-reactivity (*SI Appendix*, Fig. S3*E*). The degree of competition with hACE2 correlated with the antibody neutralizing activity as measured by a pseudovirus assay. IgG1 ab1 exhibited the highest degree of SARS-CoV-2 pseudovirus neutralization and competition with ACE2 followed by IgG1 ab2, while the hACE2-noncompeting antibodies did not show any neutralizing activities (*SI Appendix*, Fig. S3*F*). Thus, IgG1 ab1 was selected for further extensive characterization.

### IgG1 ab1 Bound with High-Affinity/Avidity to the SARS-CoV-2 RBD, S1 and Cell Surface-Associated S Protein but Not to SARS-CoV S1, and Strongly Competed with the Receptor hACE2.

**Fig. 1.**
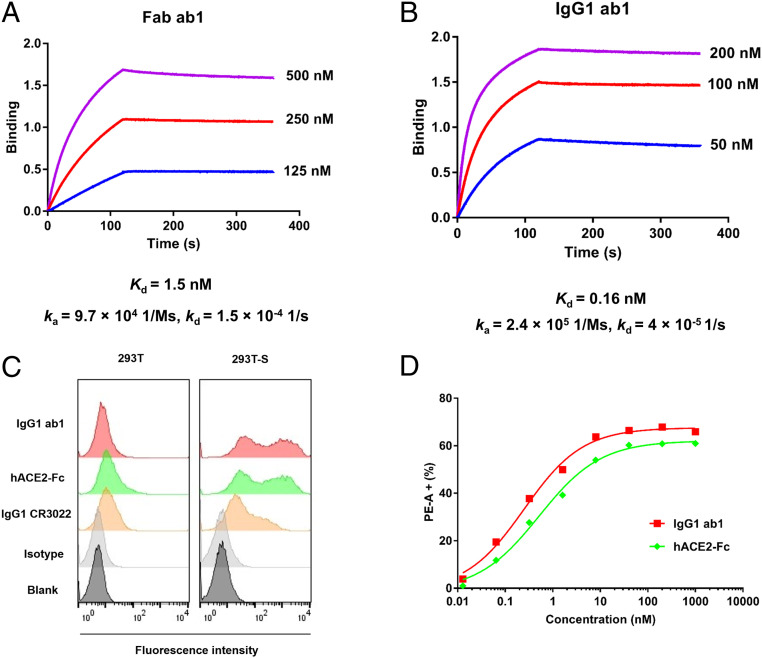
Binding kinetics of ab1 to SARS-CoV-2 RBD and cell surface-associated S. (*A*) BLItz sensorgrams for Fab ab1 binding to RBD-Fc. (*B*) Sensorgrams for IgG1 ab1 binding to RBD-Fc. (*C*) Binding of IgG1 ab1, hACE2-Fc, and IgG1 CR3022 to S transiently transfected 293T cells. The 293T cells without transfection serve as a control. Antibodies or proteins were evaluated at concentration of 1 μM. (*D*) Concentration-dependent binding of IgG1 ab1 and hACE2-Fc to 293T-S cells.

The Fab and IgG1 ab1 bound strongly to the SARS-CoV-2 RBD (*SI Appendix*, Fig. S4*A*) and S1 protein (*SI Appendix*, Fig. S4*B*) as measured by ELISA. The Fab ab1 equilibrium dissociation constant, *K*_d_, as measured by the biolayer interferometry technology (BLItz), was 1.5 nM (Fig. 1*A*). The IgG1 ab1 bound with high (160 pM) avidity to recombinant RBD (Fig. 1*B*). IgG1 ab1 bound cell surface-associated native S glycoprotein, suggesting that the conformation of its epitope on the RBD in isolation is close to that in the native S protein (Fig. 1*C*). The binding of IgG1 ab1 was of higher avidity than that of hACE2-Fc (Fig. 1*D*). Binding of IgG1 ab1 was specific for the SARS-CoV-2; it did not bind to the SARS-CoV S1 (*SI Appendix*, Fig. S3*E*) nor to cells that do not express SARS-CoV-2 S glycoprotein (Fig. 1*C*). IgG1 ab1 strongly competed with hACE2-Fc as confirmed by the BLItz (*SI Appendix*, Fig. S4*C*), and did not compete with the CR3022 (*SI Appendix*, Fig. S4*D*) which cross-reacts to SARS-CoV (16) by binding to the conserved regions in the core RBD domain distal from the receptor binding motif (RBM). The high degree of competition with hACE2 and the lack of competition with CR3022 indicate that the ab1 epitope is likely located in the RBM.

### IgG1 ab1 Potently Neutralized Authentic SARS-CoV-2 and Induced Antibody-Dependent Cellular Cytotoxicity (ADCC) in Tissue Cultures.

**Fig. 2.**
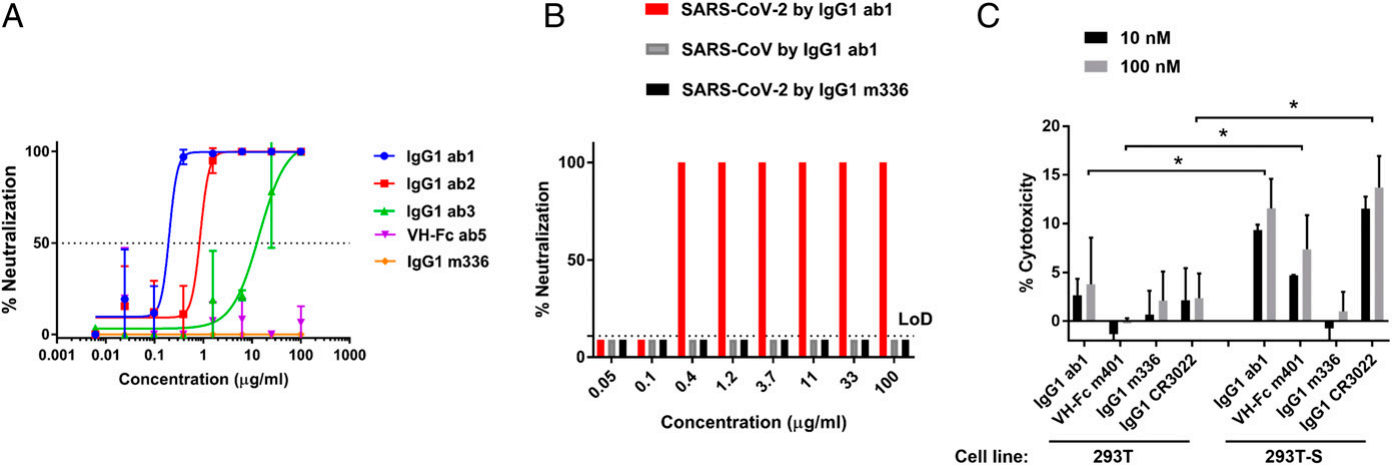
IgG1 ab1 potently neutralizes SARS-CoV-2 live virus measured by two different assays and mediates ADCC. (*A*) Neutralization of live SARS-CoV-2 by a reporter gene assay. (*B*) Neutralization of live virus by a microneutralization assay. (*C*) ADCC activity of IgG1 ab1 and VH-Fc m401 as measured by using primary human NK cells. The 293T cells overexpressing SARS-CoV-2 S were used as target cells. The cell death was monitored by using Promega LDH-Glo cytotoxicity assay. The data were analyzed by the unpaired, two-tailed, Student’s *t* test using GraphPad Prism 7.0. A *P* value <0.05 was considered significant. *P < 0.05.

IgG1 ab1 neutralized replication-competent SARS-CoV-2 significantly more potently (half-maximal inhibitory concentration, IC_50_ = 200 ng/mL) than IgG1 ab2 and IgG1 ab3 (IC_50_ = 800 ng/mL and 15 µg/mL, respectively) (Fig. 2*A*) as measured by a luciferase reporter gene assay. Because of possible variations between in vitro assays, the IgG1 ab1 neutralization activity was also tested in a different laboratory by a microneutralization (MN)-based assay, which showed similar results with a neutralization titer to achieve 100% neutralization (NT_100_) at 400 ng/mL and NT_0_ at 100 ng/mL (Fig. 2*B*). In agreement with the specificity of binding to the SARS-CoV-2 and not to the SARS-CoV, the IgG1 ab1 did not neutralize live SARS-CoV (Fig. 2*B*). The IgG1 m336 (4), which is a potent neutralizer of MERS-CoV did not exhibit any neutralizing activity against SARS-CoV-2 (Fig. 2 *A* and *B*). The correlation between virus neutralization activity and competition with hACE2 suggests that blocking of the virus S glycoprotein binding to the host receptor (hACE2) is the underlying mechanism of viral neutralization as reported for many antibodies isolated from COVID-19 patients (17–22), although some hACE2-noncompeting antibodies including 47D11 (23) and S309 (24) also exhibit neutralizing activity.

Importantly, IgG1 ab1 as well as an antibody, VH-Fc m401, which does not compete with hACE2 and does not neutralize pseudovirus (*SI Appendix*, Fig. S3 *B* and *F*), mediated ADCC although at moderate levels (10 to 15% cell killing) (Fig. 2*C*). Consistent with a recent finding (25), IgG1 CR3022 also mediated ADCC and served as a positive control (Fig. 2*C*). Such moderate levels of ADCC for IgG1s targeting the SARS-CoV-2 RBD have also been observed by others (24, 26). Antibodies with nonoverlapping epitopes, such as ab1 and m401, mediating effector functions could be potentially combined to increase efficacy and decrease the probability for escape mutants. ADCC as well as other effector functions may contribute to the control of virus infection in vivo in addition to virus neutralization but they could also lead to greater cytopathicity (25).

### IgG1 ab1 Was Highly Effective Prophylactically in Two Different Mouse Models.

**Fig. 3.**
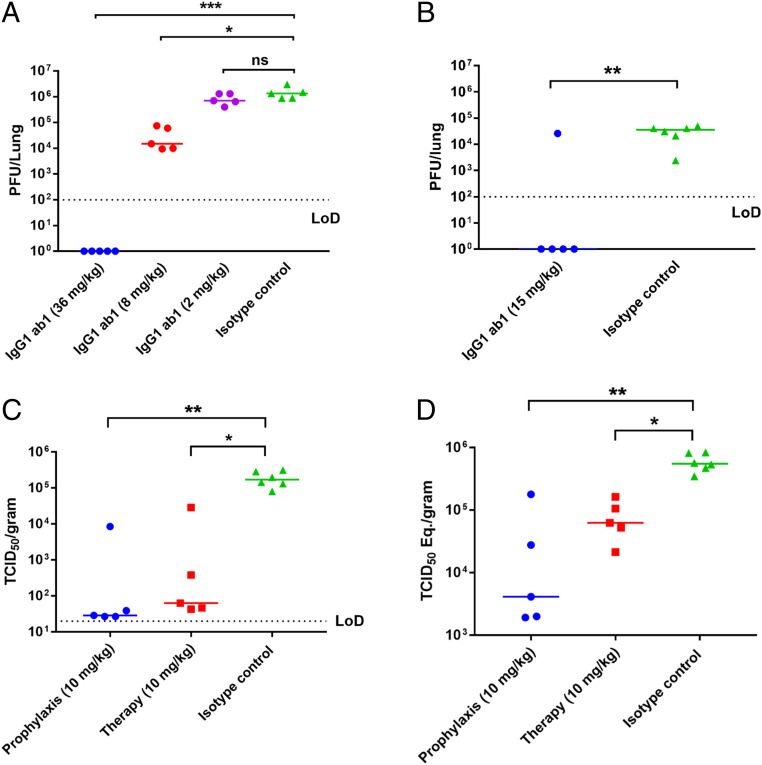
IgG1 ab1 potently neutralizes SARS-CoV-2 in three animal models. (*A*) IgG1 ab1 inhibits mouse ACE2-adapted SARS-CoV-2 in wild-type BALB/c mice. Mice were treated i.p. with varying doses of IgG1 ab1 or an isotype control 12 h prior to intranasal infection with 10^5^ pfu of mouse-adapted SARS-CoV-2. Lung tissue was homogenized in PBS and virus replication assessed by plaque assay using VeroE6 cells (Kruskal–Wallis test followed by Dunn’s test, ns: *P* > 0.05, *P < 0.05, ***P < 0.001). (*B*) IgG1 ab1 protects hACE2 transgenic mice from SARS-CoV-2 infection. The experimental protocol is similar to the one above except that human ACE2 transgenic mice and wild type SARS-CoV-2 were used (Mann–Whitney *U* test, *P < 0.05). (*C* and *D*) Evaluation of prophylactic and therapeutic efficacy of IgG1 ab1 in a hamster model of SARS-CoV-2 infection. IgG1 ab1 significantly reduced the lung viral titers (*C*) and viral RNA presented as TCID_50_ equivalents (*D*). Hamsters were injected intraperitoneally with 10 mg/kg of IgG1 ab1 antibody either 1 d before (prophylaxis) or 6 h after (therapy) intranasal challenge of 1 × 10^5^ TCID_50_ of SARS-CoV-2. At the time of killing (5 dpi), lungs were collected for virus titration by viral TCID_50_ assays and viral RNA quantification by RT-qPCR (Kruskal–Wallis test followed by Dunn’s test, *P < 0.05, **P < 0.01).

To evaluate the efficacy of IgG1 ab1 in vivo we used two mouse models of SARS-CoV-2 infection each with unique features. The first one is based on the recently developed mouse ACE2-adapted SARS-CoV-2 which has two mutations Q498T/P499Y at the ACE2 binding interface on RBD and allows the use of wild-type mice that are widely available (27). IgG1 ab1 protected mice from high titer intranasal SARS-CoV-2 challenge (10^5^ pfu) of BALB/c mice in a dose-dependent manner (Fig. 3*A*). There was complete neutralization of infectious virus at the highest dose of 36 mg/kg, statistically significant reduction by 100-fold at 8 mg/kg (Kruskal–Wallis test, *P* = 0.039), and on average 1.8-fold decrease at 2 mg/kg. The IgG1 m336 which potently neutralizes the MERS-CoV in vivo was used as an isotype control because it did not have any effect in vitro. These results also suggest that the double mutations Q498T/P499Y on RBD do not affect IgG1 ab1 binding. The second model based on transgenic mice expressing hACE2 (28) allows the use of replication-competent virus isolated from humans. Mice were administered 15 mg/kg of IgG1 ab1 prior to wild-type SARS-CoV-2 challenge followed by detection of infectious virus in lung tissue 2 d later. Replication-competent virus was not detected in four of the five mice which were treated with IgG1 ab1 (Fig. 3*B*). All six control mice and one of the treated mice had more than 10^3^ pfu per lung; the antibody probably was not transferred to the lungs in the outlier mouse. Interestingly, in both models about the same dose of antibody (10 to 15 mg/kg) reduced about 100-fold the infectious virus in the lungs. This result suggests that the evaluation of the antibody efficacy is robust in both models and supports using the mouse-adapted virus model for evaluation of inhibitors. The effective prophylactic dose of IgG1 ab1 (>2 mg/kg) is in the range (10 to 50 mg/kg) of that of other potent SARS-CoV-2 neutralizing antibodies (17, 18, 20, 21).

### IgG1 ab1 Exhibited Both Prophylactic and Therapeutic Efficacy in a Hamster Model of SARS-CoV-2 Infection.

**Fig. 4.**
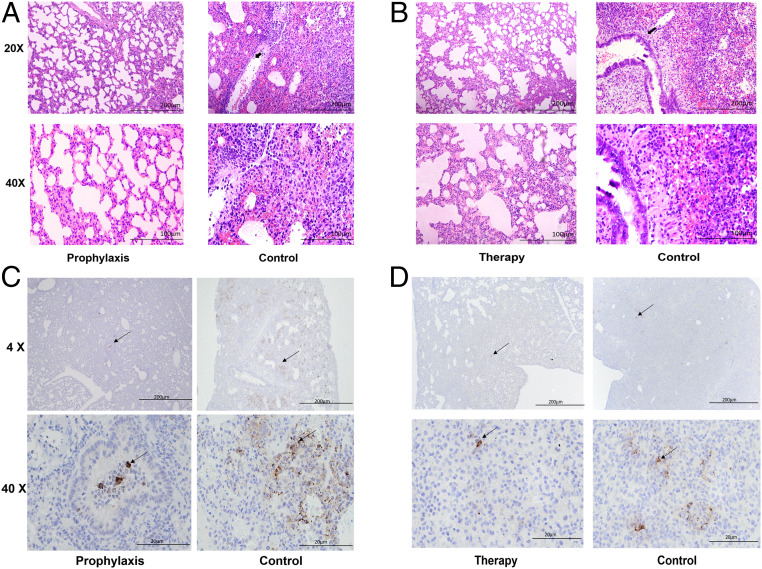
Histopathology (H&E) and IHC of hamster lung tissue. (*A* and *B*) Treatment with IgG1 ab1 reduces pathological changes in lung tissue. H&E-stained sections of lungs were compared between untreated hamsters (control), IgG1 ab1 prophylactically treated hamsters (*A*), and therapeutically treated hamsters (*B*). Images represent pathological changes in lung tissues. Arrows show the inflammatory cell infiltration with alveolar hemorrhage. (*C* and *D*) IHC for detection of SARS-CoV2 nucleocapsid antigen with anti-nucleocapsid rabbit polyclonal antibodies followed by the horseradish peroxidase (HRP)-conjugated anti-rabbit antibody. A granular, multifocal distribution is noted in lung tissue background from control animals while prophylactic treatment with IgG1 ab1 resulted in a marked reduction in the distribution of antigen-positive cells. Arrow indicates nucleocapsid-positive cells (brown) in lungs at day 5 postinfection. (*D*) The lung IHC for IgG1 ab1 therapeutically treated hamsters compared to those of controls.

**Fig. 5.**
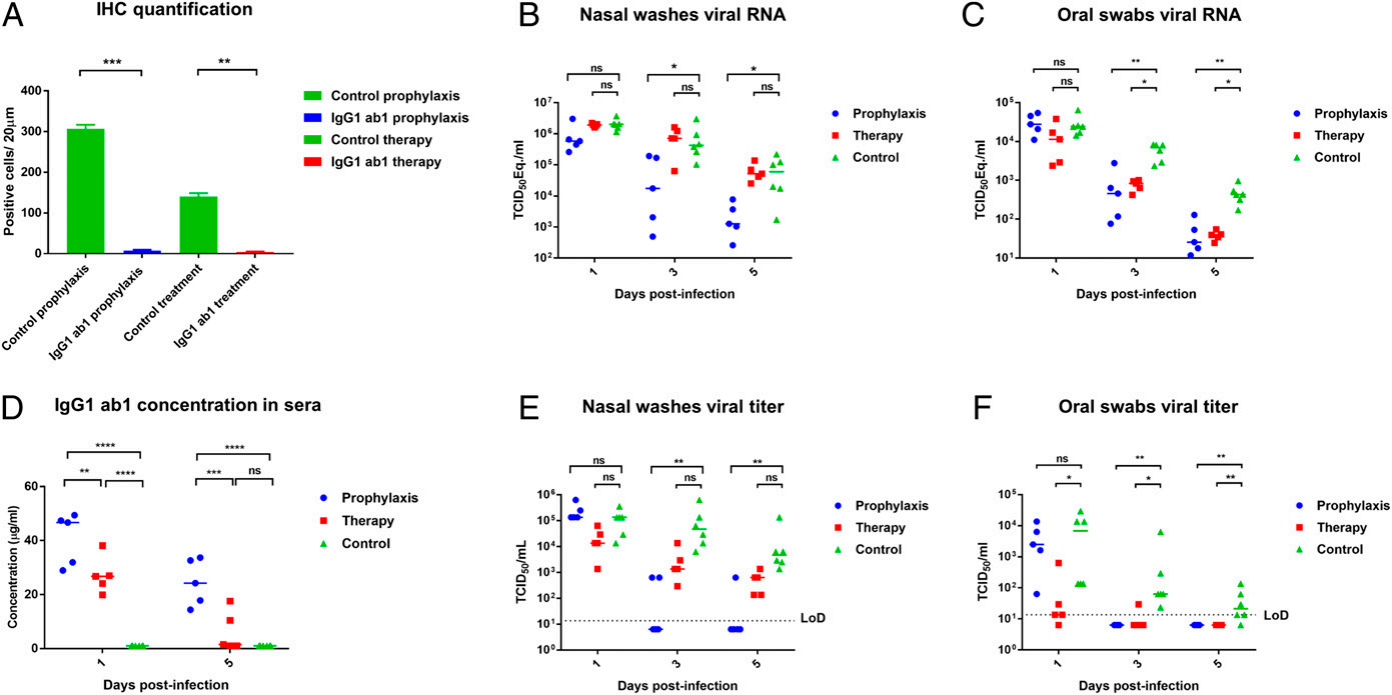
Quantification of IHC, measurement of IgG1 ab1 concentration in hamster sera postvirus challenge, and detection of infectious virus and viral RNA in hamster shedding including nasal washes and oral swabs. (*A*) Quantification of IHC image. IHC was quantified using ImageJ software by counting positive cells at 40× magnification (unpaired, two-tailed Student’s *t* test. **P < 0.01, ***P < 0.001). (*B*, *C*, *E*, and *F*) Detection of infectious virus and viral RNA in hamster nasal washes and oral swabs. Nasal washes and oral swabs were collected at day 1, 3, and 5 postinfection (dpi) for virus titer titration by TCID_50_ assays and viral RNA quantification by RT-qPCR. (*B* and *E*) Nasal washes viral RNA and viral titer in untreated, pretreated, and posttreated hamsters (Kruskal–Wallis followed by Dunn’s test, ns: *P* > 0.05, *P < 0.05, **P < 0.01). (*C* and *F*) Oral swab viral RNA and viral titer in untreated, pretreated, and posttreated hamsters (Kruskal–Wallis followed by Dunn’s test, ns: *P* > 0.05, *P < 0.05, **P < 0.01). (*D*) IgG1 ab1 concentration in hamster sera when administered prophylactically and therapeutically. Hamsters were bled at 1 and 5 dpi for measuring antibody concentrations in sera by SARS-CoV-2 S1 ELISA (two-way ANOVA followed by Tukey’s test, ns: *P* > 0.05, **P < 0.01, ***P < 0.001, ****P < 0.001).

We also used the recently developed hamster model of SARS-CoV-2 infection (29, 30) that allowed evaluation of both prophylactic and therapeutic efficacy of IgG1 ab1, although it requires a larger amount of antibody than the mouse models. Intraperitoneal (i.p.) administration of 10 mg/kg IgG1 ab1 1 d before intranasal challenge of 10^5^ 50% tissue culture infectious doses (TCID_50_) virus reduced infectious virus titer in the lungs about 10,000-fold to almost undetectable levels in four out of five hamsters at day 5 postinfection (dpi) (Fig. 3*C*). The lung viral RNA was decreased by 100-fold (Fig. 3*D*), which is similar to the decrease achieved by the neutralizing antibody CC12.1 (21). Importantly, i.p. administration of IgG1 ab1 of the same dose of 10 mg/kg 6 h after viral challenge also decreased infectious virus titer (about 3,000-fold) which is about 3-fold lower than when administered prophylactically (Fig. 3*C*); viral RNA in the lung was also decreased about 10-fold (Fig. 3*D*). The antibody was administered 6 h postviral challenge based on previous studies of SARS-CoV growth kinetics in VeroE6 cells showing a replication cycle of 5- to 6-h duration (31). IgG1 ab1 also reduced lung pathology and decreased viral antigen in the lung (Fig. 4 *A*–*D*). Hematoxylin and eosin (H&E) stain of lung tissues showed that IgG1 ab1 treatment remarkably decreased pulmonary congestion, alveolar septal thickening, and hyaline membrane formation caused by the SARS-CoV-2 infection. The H&E images were scored by a trained pathologist based on inflammation area and alveolar hemorrhage (clinical score 0, no microscopic lesions; 1, mild interstitial pneumonia; 2, moderate multifocal interstitial pneumonia; 3, moderate diffuse interstitial pneumonia; and 4, severe interstitial pneumonia). For the IgG1 ab1 prophylactic and therapeutic groups the clinical scores were equal to 1 and 2, respectively, and the control one was equal to 4. In addition, the anti-SARS-CoV-2 nucleocapsid immunohistochemistry (IHC) showed a marked reduction of antigen-positive cells in IgG1 ab1 prophylactic and therapeutic treatment groups compared to the control groups (Fig. 5*A*).

IgG1 ab1 not only decreased viral burden in the hamster lung, but also reduced viral shedding in hamster nasal washes and oral swabs (Fig. 5 *B*, *C*, *E*, and *F*). In control hamsters (infected but not treated), viral load in nasal washes was higher than that in oral swabs, and viral shedding waned faster in oral swabs, which may relate to the relatively high ACE2 expression in nasal epithelial cells and emphasizes the roles of the nasal epithelium in the initial viral infection and transmission (32). Both IgG1 ab1 prophylactic and therapeutic treatment decreased viral RNA and infectious viral titers in nasal washes and oral swabs at 3 and 5 dpi except viral RNA in the nasal washes, which was not decreased in the therapeutic group. The viral reduction at 1 dpi was not as significant as that at 3 and 5 dpi, likely due to the infection peak occurring before day 3 as reported in hamsters (33). The prophylactic treatment decreased viral loads more effectively than the therapeutic treatment. Overall, the viral RNA decrease in hamster shedding was not as obvious as the decrease observed in the lung tissue, consistent with a recent finding in hamsters (30). The decreased viral shedding in the upper airways could potentially reduce transmission of SARS-CoV-2. Here we report the results of a human mAb tested prophylactically in three different animal models, suggesting approximate equivalency of those models in terms of antibody efficacy evaluation.

Our results suggest that IgG1 ab1 can suppress the spread of newly produced virus in vivo, although the efficacy was lower compared to the prophylactic administration. Lower efficacy of therapy compared to prophylaxis was also observed for two other anti-SARS-CoV-2 antibodies (17, 20). One possible reason for the lower efficacy could be the larger amount of infectious virus produced in the animal after the first cycle(s) of replication and possibility for cell-to-cell spread. Another related contributing factor could be the decreased antibody concentration due to formation and removal of antigen/antibody complexes as we previously showed for HIV-1 (34). Indeed, the IgG1 ab1 concentration in the therapeutic group (20 to 30 μg/mL at day 1 and 0 to 15 μg/mL at day 5 after challenge) was significantly lower than that in the prophylactic group (30 to 50 μg/mL and 15 to 30 μg/mL, respectively) (Fig. 5*D*). Similar concentrations were reported for the neutralizing antibody CC12.1 (21). The IgG1 ab1 concentration in sera needed for protection was much higher (∼250-fold) than the in vitro live virus IC_50_ which is generally observed for many antiviral antibodies (35). The relatively high concentration of IgG1 ab1 6 d after administration also indicates good pharmacokinetics.

### IgG1 ab1 Has Relatively Low Levels of Somatic Hypermutations and Good Developability.

Interestingly, Fab ab1 had only several somatic mutations compared to the closest germline predecessor genes, which was also observed for many neutralizing antibodies from COVID-19 patients (36–38). We and others have demonstrated that germline-like antibodies can also be highly effective against other viruses causing acute infections such as henipaviruses (5, 6), SARS-CoV (7), MERS-CoV (39), influenza (40), Dengue virus (41) and Zika virus (42); they can be rapidly elicited through an “innate-like” antiviral recognition mediated by antigen-specific naive B cell receptors in a germinal center-independent manner (43). The low number of somatic hypermutations of ab1 implies that ab1-like antibodies could be elicited relatively quickly by using RBD-based immunogens especially in some individuals with naive mature B cells expressing the germline predecessors of ab1. This is in contrast to the highly mutated broadly neutralizing HIV-1 antibodies that require long maturation times, are difficult to elicit, and their germline predecessors cannot bind native HIV-1 envelope glycoproteins (44, 45). The germline-like nature of the newly identified mAb ab1 also indicates that it has excellent developability properties that could accelerate its development for prophylaxis and therapy of SARS-CoV-2 infection (46).

To further assess the developability (druggability) of ab1, its sequence was analyzed online (http://opig.stats.ox.ac.uk/webapps/newsabdab/sabpred/tap); no obvious liabilities were found. In addition, we used dynamic light scattering (DLS) and size exclusion chromatography (SEC) to evaluate its propensity for aggregation. IgG1 ab1 at a concentration of 2 mg/mL did not aggregate after 6 days of incubation at 37 °C as measured by DLS (*SI Appendix*, Fig. S5*A*); there were no high molecular weight species in freshly prepared IgG1 ab1 also as measured by SEC (*SI Appendix*, Fig. S5*B*). IgG1 ab1 also did not bind to the human cell line 293T (Fig. 1*C*) even at very high concentration (1 μM) which is about 660-fold higher than its *K*_d_, indicating absence of nonspecific binding to many membrane-associated human proteins. The IgG1 ab1 also did not bind to 5,300 human membrane-associated proteins as measured by a membrane proteome array (*SI Appendix*, Fig. S5*C*).

## Conclusion

The high affinity/avidity and specificity of IgG1 ab1 along with potent neutralization of virus and good developability properties suggest its potential use for prophylaxis and therapy of SARS-CoV-2 infection. Because it strongly competes with hACE2 indicating a certain degree of mimicry, one can speculate that mutations in the RBD that decrease ab1 binding may also lead to inefficient entry into cells and infection. However, in the unlikely case of such mutations, ab1 can be used in combination with other mAbs with distinct epitopes including those we identified here or in bi(multi)specific formats. Ab1 could also be used to select appropriate epitopes for vaccine immunogens and for diagnosis of SARS-CoV-2 infections. The identification of neutralizing mAbs within days of target availability shows the potential value of large antibody libraries for rapid response to emerging viruses.

## Methods

### Generation of SARS-CoV-2 RBD, Panning of Phage Libraries, and Screening by ELISA and BLItz.

SARS-CoV-2 RBD-his and Fc, S1-Fc, ACE2-Fc, CR3022 Fab, and IgG1 were subcloned into pcDNA3.1. Proteins were expressed with the Expi293 expression system and purified with protein A resin or by Ni-NTA resin. The recombinant RBD proteins was used to pan our naive human antibody phage display libraries, which were made based on the antibody cDNA from a total of 490 healthy donors’ PBMCs and splenocytes. These libraries contain very large transformants (size for each ∼10^11^) and are highly diverse. Biopanning was based on the pull-down method by using streptavidin-M280 Dynabeads. After panning, positive binders were selected by phage ELISA. Their binding was subsequently measured by RBD binding ELISA, hACE2 competition ELISA, and the binding kinetics were measured by the biolayer interferometry technology (BLItz). The leading candidates were converted to the IgG1 or VH-Fc fusion formats.

### Neutralization of Pseudotyped and Replication-Competent SARS-CoV-2 and In Vitro ADCC Assay.

The pseudovirus neutralization assay was based on the SARS-CoV-2 S pseudotyped HIV-1 virus (with luciferase in the genome) entry into hACE2-expressing cells. For testing neutralization against live SARS-CoV-2, we used two independent assays. The first one is the standard live virus-based MN assay based on the microscopic observation of virus-induced formation of cytopathic effect. The other one is based on the full-length viruses expressing luciferase, which were designed and recovered via reverse genetics and described previously (47). For the ADCC assay, human natural killer (NK) cells from healthy donors were isolated from PBMCs. The 293T cells stably expressing SARS-CoV-2 S (293T-S) were used as target cells. Cell death was evaluated by using the LDH-Glo cytotoxicity assay.

### Evaluation of IgG1 ab1 Prophylactic and Therapeutic Efficacy in Three Animal Models.

For the inhibition of mouse-adapted SARS-CoV-2 in wild-type mice, a recombinant mouse ACE2-adapted SARS-CoV-2 virus was constructed (27). Groups of 5 each of 10- to 12-mo-old female BALB/c mice were treated prophylactically (12 h before 10^5^ pfu intranasal infection) intraperitoneally with doses of 36, 8, and 2 mg/kg. Two days postinfection, mice were killed, and lung viral titer was determined by plaque assay. For the evaluation of IgG1 ab1 efficacy in the hACE2 mouse model, hACE2 transgenic 6- to 9-wk-old C3B6 mice were treated intraperitoneally with 0.3 mg (15 mg/kg) of antibody (five mice) or negative controls (six mice) 15 h prior to intranasal infection with 10^5^ pfu of wild-type SARS-CoV-2. Lung tissue was homogenized in phosphate-buffered saline (PBS) and virus replication assessed by plaque assay on VeroE6 cells. In the hamster model of SARS-CoV-2 infection, all hamsters (*n* = 5) were injected intraperitoneally with 10 mg/kg of IgG1 ab1 antibody either 24 h prior to (prophylaxis) or 6 h after (therapy) intranasal challenge of 1 × 10^5^ TCID_50_ of SARS-CoV-2. Untreated hamsters were kept as a control. Nasal washes and oral swabs were collected at days 1, 3, and 5 postinfection. Hamsters were bled at 1 and 5 dpi. All hamsters were killed on 5 dpi. At the time of killing, lungs were collected for virus titration and RNA isolation. For testing sera IgG1 ab1 concentrations, SARS-CoV-2 spike-1 (S1) ELISA was used. For histopathology on day 5 postinfection, 10% formalin-fixed and paraffin-embedded tissues were processed with either H&E or IHC for detection of SARS-CoV2 nucleocapsid antigen. Lung lobe H&E-stained images were scored based on pathology using microscopy. IHC was quantified using ImageJ software by counting positive cells at 40× magnification.

Detailed materials and methods for this study are described in *SI Appendix*.

### Ethics Statement.

Human ACE2 transgenic C3B6 mice (6 to 9 wk old) and BALB/c mice (10 to 12 wk old) were used for all experiments. The study was carried out in accordance with the recommendations for care and use of animals by the Office of Laboratory Animal Welfare, National Institutes of Health, and the Institutional Animal Care and Use Committee of the University of North Carolina (UNC permit no. A-3410-01). For the hamster model, studies were approved by the University Animal Care Committee of the University of Saskatchewan according to the guidelines of the Canadian Council on Animal Care.

### Statistical Analyses.

Statistics of the ADCC and IHC quantification data were determined by the unpaired, two-tailed, Student’s *t* test using GraphPad Prism 7.0; *P < 0.05, **P < 0.01, ***P < 0.001. The hACE2 transgenic mouse data were analyzed by the Mann–Whitney *U* test; *P < 0.05. The significances for the mouse ACE2-adapted model and viral titer, viral RNA in hamsters lung, nasal washes, and oral swabs were determined by the Kruskal–Wallis test followed by Dunn’s test; ns: *P* > 0.05, *P < 0.05, **P < 0.01, ***P < 0.001. The significance of IgG1 ab1 concentration in hamster sera was determined by the two-way ANOVA analysis followed by Tukey’s test; ns: *P* > 0.05, **P < 0.01, ***P < 0.001, ****P < 0.0001.

## Supplementary Material

Supplementary File

## Data Availability

All data supporting the findings of this study are included in the main text and *SI Appendix*; physical materials will be made available upon request after completion of a Material Transfer Agreement. Antibody variable domain sequences were deposited to GenBank with accession numbers MW118116 and MW118117 and are only allowed for noncommercial use.
