# Supplementary material for: Rapid identification of a human antibody with high prophylactic and therapeutic efficacy in three animal models of SARS-CoV-2 infection
Source: Proc Natl Acad Sci U S A. 2020 Nov 2;117(47):29832–8. doi: 10.1073/pnas.2010197117 (PMC7703590; doi:10.1073/pnas.2010197117)
Supplement: Supplementary File [file pnas.2010197117.sapp.pdf]

Supplementary Information for:

Rapid identification of a human antibody with high prophylactic and therapeutic efficacy in three animal models of SARS-CoV-2 infection

Wei Li[a#*], Chuan Chen[a#], Aleksandra Drelich[b#], David R. Martinez[c#], Lisa Gralinski[c#], Zehua Sun[a#], Alexandra Schäfer[c#], Swarali S. Kulkarni[d#], Xianglei Liu[a], Sarah R. Leist[c], Doncho Zhelev[a], Liyong Zhang[a], Ye-Jin Kim[a], Eric C. Peterson[e], Alex Conard[e], John W. Mellors[a,e], Chien-Te Tseng[b], Darryl Falzarano[d], Ralph S. Baric[c] and Dimiter S. Dimitrov[a,e*]

Center for Antibody Therapeutics, Division of Infectious Diseases, Department of Medicine, University of Pittsburgh Medical School, 3550 Terrace Str, Pittsburgh, PA 15261, USA[a]; Department of Microbiology & Immunology, Centers for Biodefense and Emerging Diseases, Galveston National Laboratory, 301 University Blvd, Galveston, Texas 77550, USA[b]; University of North Carolina at Chapel Hill, 135 Dauer Drive, 3109 Michael Hooker Research Center Chapel Hill, NC 27599[c]; Vaccine and Infectious Disease Organization – International Vaccine Centre, and the Department of Veterinary Microbiology, University of Saskatchewan, 117 Veterinary Road, Saskatoon, SK S7N 5E3, Canada[d]; Abound Bio, 1401 Forbes Ave, Pittsburgh, PA 15219[e]

Running title: An antibody effective in animal models of SARS-CoV-2

***Corresponding author:** Dimiter S. Dimitrov; **Email:** mit666666@pitt.edu

***Second corresponding author:** Wei LI; **Email:** liwei171@pitt.edu

[#] **Equal contribution**

This PDF file includes:

Materials and Methods
Figures and Figure Legends for S1 to S5
SI references

www.pnas.org/cgi/doi/10.1073/pnas.2010197117

Supplementary Information Text

Materials and Methods

Generation, Expression and Characterization of SARS-CoV-2 RBD-Fc, S1-Fc, ACE2-Fc and CR3022 Fab. The SARS-CoV-2 surface glycoprotein and the anti-SARS-CoV antibody IgG1 CR3022 (1) were synthesized by IDT (Coralville, Iowa). MERS-CoV-specific IgG1 m336 antibody was expressed in human mammalian cell as described previously (2). The ACE2 gene was ordered from OriGene (Rockville, MD). The RBD domain (residues 330-532) and S1 domain (residues 14-675) and ACE2 (residues 18-740) genes were cloned into plasmid which carries a CMV promotor with an intron, human IgG1 Fc region and Woodchuck posttranscriptional regulatory element (WPRE) to generate the RBD-Fc, S1-Fc and ACE2-Fc expression plasmids. The RBD-avi-his protein with an avi tag followed by a 6×His tag at C-terminal was subcloned similarly. These proteins were expressed with Expi293 expression system (Thermo Fisher Scientific) and purified with protein A resin (GenScript) and by Ni-NTA resin (Thermo Fisher Scientific). The Fab CR3022 antibody gene with a His tag was cloned into pCAT2 plasmid (developed in house) for expression in HB2151 bacteria and purified with Ni-NTA resin. Protein purity was estimated as >95% by SDS-PAGE and protein concentration was measured spectrophotometrically (NanoVue, GE Healthcare).

Selection, Expression, and Purification of the RBD-specific Fabs and VHs and Conversion to IgG1s or Fc Fusion Proteins. The naïve human antibody phage display libraries were made based on the antibody cDNA from total of 490 healthy donors peripheral blood monocytes (PBMCs) and splenocytes. The Fab and scFv libraries were

constructed by randomly pairing antibody VH and VL gene, and the VH libraries - by grafting CDRs into stable VH scaffolds. These libraries contain very large transformants (size for each ~10^{11}) and are highly diverse. For panning, the libraries were preabsorbed on streptavidin-M280-Dynabeads in PBS for 1 h at room temperature (RT) and incubated with 50 nM biotinylated SARS-CoV-2 RBD for 2 h at room temperature with gentle agitation. Phage particles binding to biotinylated antigen were separated from the phage library using streptavidin-M280-Dynabeads and a magnetic separator (Dynal). After washing for 20 times with 1 ml of PBS containing 0.1% Tween-20 and another 20 times with 1 ml of PBS, bound phage particles were eluted from the beads using 100 mM triethanolamine followed by neutralization with 1 M, pH 7.5 Tris-HCl. For the 2nd round of panning, 10 nM (2 nM for the 3rd round) of biotinylated antigen was used as antigen. After the 3rd round of panning against 2 nM biotinylated antigen, 96 individual clones were screened for binding to RBD-Fc fusion protein by phage ELISA. Panels of Fabs and VHs were selected and sequenced. For conversion to Fc-fusion, the VH gene was subcloned into pSecTag B vector (already containing human Fc fragment). For conversion to IgG1, Fab VH and VL gene was inserted into pDR12 vector which contains the IgG1 CH1-CH3 and CL domains. Both VH-Fc and IgG1 were expressed as previously described (3). Protein purity was estimated as >95% by SDS-PAGE and protein concentration was measured spectrophotometrically (NanoVue, GE Healthcare).

ELISA. For phage ELISA, the SARS-CoV-2 RBD-Fc (residues 330-532) protein was coated on a 96-well plate (Costar, half-area, #3690) at 100 ng/well in PBS overnight at 4°C. Phage from each round of panning (polyclonal phage ELISA) or clones randomly picked from the infected TG1 cells (monoclonal phage ELISA) were incubated with

immobilized antigen. Bound phage were detected with horseradish peroxidase (HRP) conjugated anti-M13-HRP polyclonal Ab (Pharmacia, Piscataway, NJ). For the soluble Fab/VH binding assay, 200 ng RBD-Fc was coated and HRP-conjugated mouse anti-FLAG tag Ab (Sigma-Aldrich) was used to detect Fab/VH binding. For the IgG1 or VH-Fc binding assay, 200 ng RBD-his was coated and HRP-conjugated goat anti-human IgG Fc (Sigma-Aldrich) was used for detection. For the competition ELISA with hACE2, 2 nM of human ACE2-mouse Fc (Sino Biological) was incubated with plate-coated RBD-Fc in the presence of serially diluted IgG1 or VH-Fc. After washing, bound ACE2-mouse Fc was detected by HRP-conjugated anti mouse IgG (Fc specific) (Sigma-Aldrich). For the competition ELISA between ab1 and other antibodies, ~20 nM Fab ab1 was incubated with RBD-Fc in the presence of different concentrations of antibodies in IgG1 or VH-Fc formats. After washing, detection was made by using HRP conjugated anti-FLAG tag antibody. For the competition ELISA with CR3022, 10 nM Fab CR3022 was incubated with serially diluted IgG1 or VH-Fc antibodies, and the mixtures were added to RBD-Fc coated wells. After washing, bound Fab CR3022 was detected by HRP-conjugated anti-FLAG tag antibody. All the colors were developed by 3,3′,5,5′-tetramethylbenzidine (TMB, Sigma) and stopped by 1 M H_2SO_4 followed by recording absorbance at 450 nm. Experiments were performed in duplicate and the error bars denote ± SD, n =2.

BLItz. Antibody affinities and avidities were analyzed by the biolayer interferometry BLItz (ForteBio, Menlo Park, CA). For affinity measurements, protein A biosensors (ForteBio: 18–5010) were coated with RBD-Fc for 2 min and incubated in DPBS (pH = 7.4) to establish baselines. 125 nM, 250 nM and 500 nM Fab ab1 were used for association. For avidity measurements, RBD-Fc was biotinylated with EZ-link sulfo-NHS-LC-biotin

(Thermo Fisher Scientific, Waltham, MA) (RBD-Fc-Bio). Streptavidin biosensors (ForteBio: 18–5019) were coated with RBD-Fc-Bio for 2 min and incubated in DPBS (pH = 7.4) to establish baselines. 50 nM, 100 nM and 200 nM IgG1 ab1 was chosen for association. The association was monitored for 2 min and then the antibody allowed to dissociate in DPBS for 4 min. The k_a and k_d were derived from the sensorgrams fitting and used for K_d calculation. For the competitive Blitz, 500 nM IgG1 ab1 was loaded onto the RBD-Fc coated sensor for 300 s to reach saturation followed by dipping the sensor into the 100 nM hACE2-mouse Fc or Fab CR3022 solution in the presence of 500 nM IgG1 ab1. The association was monitored for 300 s. Meanwhile, the signals of 100 nM hACE2 or CR3022 binding to the RBD-Fc coated sensor in the absence of IgG1 ab1 was independently recorded. Competition was determined by the percentage of signals in the presence of ab1 to signals in the absence of ab1 (< 0.7 is considered to be competitive) (4).

Flow Cytometry Analysis. Full-length S protein of SARS-CoV-2 with native signal peptide replaced by the CD5 signal peptide were codon-optimized and synthesized by IDT. S gene was subcloned into our in-house mammalian cell expression plasmid, which were used to transiently transfect 293T cells cultured in Dulbecco's Modified Eagle's Medium (DMEM) with 10% FBS, 1% P/S. The transient expression level is tested by FACS staining using the recombinant hACE2-human Fc, IgG1 CR3022 and IgG1 ab1. For the determination of binding avidity of IgG1 ab1 and hACE2-Fc to the cell surface S, quantitative FACS was performed by using the 48 h post transfection cells based on standard procedures. Briefly, 5 folds serially diluted antibodies or hACE2-Fc with highest concentration of 1 μM were added into 1×10^6 cells and incubate at 4 °C for 30 min followed by 3 times washing using PBS + 0.5% BSA buffer (PBSA buffer). Then cells were

resuspended to 100 μl PBSA buffer followed by addition of 1 μl PE conjugated anti-human Fc antibody (Sigma-Aldrich) and incubate at 4 °C for 30 min. Cells then was washed by PBSA for 3 times and then analyzed by flow cytometry using BD LSR II (San Jose, CA). The gating of PE-A+ population was performed by FlowJo_V10_CL. The concentrations at which IgG1 ab1 or hACE2-Fc achieved 50% PE-A+ cells (EC_{50}) was obtained by non-linear fitting in the Graphpad Prism 7 (San Diego, CA).

Pseudovirus Neutralization Assay. The pseudovirus neutralization assay was based on the SARS-CoV-2 S pseudotyped HIV-1 virus entry into hACE2 expressing cells assay according to previous protocols (5). Briefly, HIV-1 backbone based pseudovirus was packaged in 293T cells by co-transfecting with plasmid encoding SARS-CoV-2 S protein and plasmid encoding luciferase expressing HIV-1 genome (pNL4-3.luc.RE) using polyethylenimine (PEI). Pseudovirus-containing supernatants were collected 48 h later and concentrated using Lenti-X™ concentrator kit (Takara, CA). Pseudovirus neutralization assay was then performed by incubation of SARS-CoV-2 pseudovirus with 5 folds serially diluted antibodies or hACE2-human IgG1 Fc (prepared in house) with starting concentration of 4 μg/ml for 1 h at 37 °C, followed by addition of the mixture into pre-seeded hACE2 expressing cells in duplicate. The mixture was then centrifuged at 1000 × g for 1 hour at room temperature. The medium was replaced 4 hrs later. After 24 h, luciferase expression was determined by Bright-Glo kits (Promega, Madison, WI). The virus only (S_{virus}) and cells (S_{blank}) only were set up for the normalization. The neutralization percentage was obtained by the equation: $(S_{virus}-S_{antibdoy})/(S_{virus}-S_{blank}) \times 100\%$. The 50% pseudovirus neutralizing antibody titer (IC_{50}) was calculated by non-linear fitting the plots of neutralization percentage against antibody concentrations in the

Graphpad Prism 7. Experiments were performed in duplicate and the error bars denote ± SD, n =2.

SARS-CoV and SARS-CoV-2 Microneutralization Assay. The standard live virus-based microneutralization (MN) assay was used as previously described (6-9). Briefly, serially three-fold and duplicate dilutions of individual monoclonal antibodies (mAbs) were prepared in 96-well microtiter plates with a final volume of 60 μl per well before adding 120 infectious units of SARS-CoV or SARS-CoV-2 in 60 μl to individual wells. The plates were mixed well and cultured at room temperature for 2 h before transferring 100 μl of the antibody-virus mixtures into designated wells of confluent Vero E6 cells grown in 96-well microtiter plates. Vero E6 cells cultured with medium with or without virus were included as positive and negative controls, respectively. Additionally, Vero E6 cells treated with the MERS-CoV RBD-specific neutralizing m336 mAb (2) were included as additional controls. After incubation at 37 ºC for 4 days, individual wells were observed under the microcopy for the status of virus-induced formation of cytopathic effect. The efficacy of individual mAbs was expressed as the lowest concentration capable of completely preventing virus-induced cytopathic effect in 100% of the wells.

SARS-CoV and SARS-CoV-2 Reporter Gene Neutralization Assay. Full-length viruses expressing luciferase were designed and recovered via reverse genetics and described previously (10, 11). Viruses were tittered in Vero E6 USAMRID cells to obtain a relative light units (RLU) signal of at least 20× the cell only control background. Vero E6 USAMRID cells were plated at 20,000 cells per well the day prior in clear bottom black walled 96-well plates (Corning 3904). MAb samples were tested at a starting dilution 100 μg/ml, and were serially diluted 4-fold up to eight dilution spots. SARS-CoV-UrbaninLuc,

and SARS-CoV-2-SeattlenLuc viruses were diluted in separate biological safety cabinets (BSC) in accordance with UNC safety rules and were mixed with serially diluted antibodies. Antibody-virus complexes were incubated at 37°C with 5% CO_2 for 1 hour. Following incubation, growth media was removed and virus-antibody dilution complexes were added to the cells in duplicate. Virus-only controls and cell-only controls were included in each neutralization assay plate. Following infection, plates were incubated at 37°C with 5% CO_2 for 48 hours. After the 48 hours incubation, cells were lysed and luciferase activity was measured via Nano-Glo Luciferase Assay System (Promega) according to the manufacturer specifications. SARS-CoV and SARS-CoV-2 neutralization IC_{50} were defined as the sample concentration at which a 50% reduction in RLU was observed relative to the average of the virus control wells. Experiments were performed in duplicate and IC_{50} was obtained by the non-linear fitting of neutralization curves in Graphpad Prism 7.

Antibody Dependent Cellular Cytotoxicity (ADCC) Assay. Human NK cells were isolated from PBMCs (purchased from Zen-Bio, NC) by using NK cells isolation kit combined with MACS (Miltenyi, Cat. No. 130-092-657). 293T cells stably expressing SARS-CoV-2 S (293T-S) were used as target cells. 293T-S cells (5×10^3 cells/well) were incubated with 10 nM and 100 nM antibodies for 30 min before addition of human NK cells (2.5×10^4 cells/well). IgG1 CR3022 and IgG1 m336 were used as positive and negative controls. After 3.5 hours incubation at 37°C, 5% CO2, the plates were equilibrated to room temperature for 10 minutes. The cell death was evaluated by using the LDH-Glo cytotoxicity assay (Promega, #J2381), which measures the LDH activity in the cell culture supernatant correlating to the cell death. The cytotoxic percentage was calculated as

follows: 100 × (experimental-effector only-target only)/(target maximum-target only, where effector only and target only represents the spontaneous background signals of NK cells and 293T-S cells. The 293T cells which do not express S proteins were also assayed to ascertain the ADCC specificity.

Inhibition of Mouse Adapted SARS-CoV-2 in Wild Type Mice. A recombinant mouse ACE2 adapted SARS-CoV-2 variant was constructed by introduction of two amino acid changes (Q498T/P499Y) at the ACE2 binding pocket in RBD (12). Virus stocks were grown on Vero E6 cells and viral titer was determined by plaque assay. Groups of 5 each of 10 to 12-month old female BALB/c mice (Envigo, #047) were treated prophylactically (12 hours before infection) intraperitoneally with 900 μg, 200 μg, or 50 μg of IgG1 ab1, respectively (corresponding to doses of 36, 8 and 2 mg/kg). Mice were challenged intranasally with 10^5 PFU of mouse-adapted SARS-CoV-2. Two days post infection, mice were sacrificed, and lung viral titer was determined by the plaque assay.

Evaluation of IgG1 ab1 Protective Efficacy in a hACE2 Mouse Model of Infection. Human ACE2 transgenic 6-9 week old C3B6 mice were treated intraperitoneally with 0.3 mg (15 mg/kg) of antibody (5 mice) or negative controls (6 mice) 15 hours prior to intranasal infection with 10^5 PFU of SARS-CoV-2. No weight loss was observed over the course of the two-day infection. Lung tissue was homogenized in PBS and virus replication assessed by plaque assay on VeroE6 cells. The assay limit of detection was 100 PFU.

Evaluation of both Prophylactic and Therapeutic Efficacy for IgG1 ab1 in a Hamster Model of SARS-CoV-2 Infection. SARS-CoV2/Canada/ON/VIDO-01/2020 was propagated on Vero'76 cells using DMEM with 2% FBS and 1μg/ml TCPK trypsin. Infectious work with SARS-CoV-2 was approved by the Biosafety Protocol Approval

Committee (BPAC) at the University of Saskatchewan and performed in the high containment laboratories at VIDO-InterVac. Male hamsters (9-week-old) were obtained from Charles River (Montreal, QC). For evaluations of prophylactic efficacy, all hamsters (n=5) were injected intraperitoneally with 10 mg/kg of IgG1 ab1 antibody 24 hours prior to intranasal challenge of 50 µl/nare containing a total of 1×10^5 50% tissue culture infectious doses ($TCID_{50}$) of SARS-CoV-2. For the therapeutic group, hamsters (n=5) were infected as above and treated intraperitoneally with 10 mg/kg of IgG1 ab1 6 hours post-infection. Untreated hamsters were kept as a control. Nasal washes and oral swabs were collected at day 1, 3 and 5 post infection (dpi). Hamsters were bled at 1 and 5 dpi. All hamsters were euthanized on 5 dpi. At euthanasia, lung lobes were collected for virus titration and RNA isolation. For viral titer determination, nasal washes were diluted in a 10-fold dilution series and absorbed on Vero'76 cells in triplicates for 1 hour at 37°C. Inoculum was removed and replaced with fresh DMEM containing 2% FBS, penn/strep and 1µg/ml TPCK. To determine virus titers from tissues, lungs were homogenized with DMEM and then processed as above. Cytopathic effect was scored on day 3 and day 5 post infection. The limit of detection is 13.6 $TCID_{50}$. For testing viral RNA, viral RNA isolated from nasal and oral swabs using the QiaAmp Viral RNA mini kit (Qiagen) and the QuantiFast Probe RT-PCR kit (Qiagen) to amplify a portion of upE gene. For RNA levels in tissues, 30 mg of tissue homogenate in RLT were processed with the RNeasy kit (Qiagen) followed by RT-qPCR as above. $TCID_{50}$ equivalence were estimated by running serial dilutions of known $TCID_{50}$ standards. For testing sera IgG1 ab1 concentrations post delivery, SARS-CoV-2 spike-1 (S1) ELISA was used. S1 protein was coated at 1 µg/ml overnight at 4°C in PBS onto MaxiSorp plates (Nunc). The following day plates were

blocked with 5% skim milk and 0.05% Tween20. Serum collected on day 1 and day 5 post-challenge was diluted 1:100 and absorbed for 1 hour at 37 °C. Plates were washed and goat anti human IgG-HRP was added. Plates were washed and subsequently developed with OPD (o-phenylenediamine dihydrochloride) substrate. Optical density was measured at 450 nm after 30 mins of incubation. For histopathology on day 5 p.i, 10% formalin fixed and paraffin embedded tissues were processed with either hematoxylin and Eosin stain (H&E) or immunohistochemistry (IHC) for detection of SARS-CoV2 antigen. Briefly, In IHC after blocking tissue slides were treated with anti-Nucleocapsid rabbit polyclonal antibodies followed with anti-rabbit HRP antibody. Lung lobes were scored based on pathology using microscopy.

Dynamic Light Scattering (DLS). For evaluation of aggregation propensity, IgG1-ab1 were buffer-changed to DPBS and filtered through a 0.22 μm filter. The concentration was adjusted to 2 mg/mL; ~500 μL samples were incubated at 37 °C. On day 0, day 1 and day 6, samples were taken out for DLS measurement on Zetasizer Nano ZS ZEN3600 (Malvern Instruments Limited, Westborough, MA) to determine the size distributions of protein particles.

Size Exclusion Chromatography (SEC). The Superdex 200 Increase 10/300 GL chromatography (GE Healthcare, Cat. No. 28990944) was used. The column was calibrated with protein molecular mass standards of Ferritin (Mr 440 000 kDa), Aldolase (Mr 158 000 kDa), Conalbumin (Mr 75 000 kDa), Ovalbumin (Mr 44 000 kDa), Carbonic anhydrase (Mr 29 000 kDa), Ribonuclease A (Mr 13 700 kDa). ~150 ul filtered proteins (1.5 mg/ml) in PBS were used for analysis. Protein was eluted by DPBS buffer at a flow rate of 0.5 ml/min.

Computational Analysis of Antibody Sequences. IMGT/V-QUEST tool (13) was used to perform immunogenetic analysis of SARS-CoV-2 RBD-specific mAbs. The unrooted circular phylogram tree of our scFv binders was constructed by using the neighbor joining methods through CLC Genomics Workbench 20.0 (https://digitalinsights.qiagen.com). The RBD sequences were obtained from https://www.ncbi.nlm.nih.gov/genbank/sars-cov-2-seqs/. Liabilities were evaluated online (opig.stats.ox.ac.uk/webapps/sabdab-sabpred/TAP.php) (14).

Membrane Proteome Array Specificity Testing Assay. Integral Molecular, Inc. (Philadelphia, PA) performed specificity testing of IgG1 ab1 using the Membrane Proteome Array (MPA) platform. The MPA comprises 5,300 different human membrane protein clones, each overexpressed in live cells from expression plasmids that are individually transfected in separate wells of a 384-well plate (15). The entire library of plasmids is arrayed in duplicate in a matrix format and transfected into HEK-293T cells, followed by incubation for 36 h to allow protein expression. Before specificity testing, optimal antibody concentrations for screening were determined by using cells expressing positive (membrane-tethered Protein A) and negative (mock-transfected) binding controls, followed by flow cytometric detection with an Alexa Fluor-conjugated secondary antibody (Jackson ImmunoResearch Laboratories). Based on the assay setup results, ab1 (20 μg/ml) was added to the MPA. Binding across the protein library was measured on an iQue3 (Ann Arbor, MI) using the same fluorescently labeled secondary antibody. To ensure data validity, each array plate contained positive (Fc-binding; SARS-CoV-2 S protein) and negative (empty vector) controls. Identified targets were confirmed in a second flow

cytometric experiment by using serial dilutions of the test antibody. The identity of each target was also confirmed by sequencing.

Supplementary Figures and Figure Legends

Figure S1

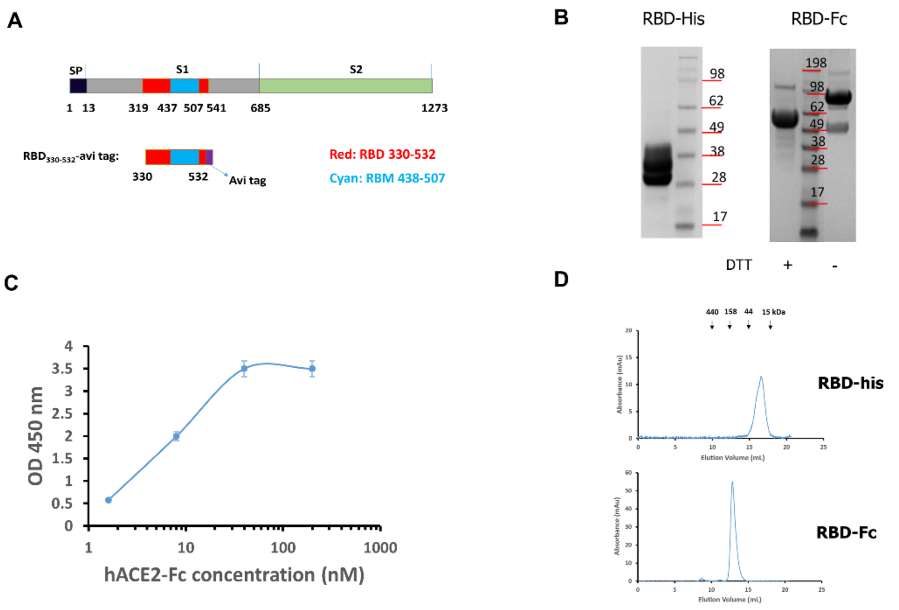

Figure S1. Schematic representation of SARS-CoV-2 S and RBD, and characterization of the RBD as an antigen for panning. (A) Schematic representation of the SARS-CoV-2 S and RBD. RBD is highlighted by the red color and the receptor-binding motif (RBM) is pictured by cyan color. $RBD_{330\text{-}532}$ is recombinantly expressed in mammalian cells with a C terminal avi tag for in vitro BirA mediated biotinylation. **(B)** SDS-PAGE of RBD-avi-his and RBD-Fc in the presence or absence of DTT. The apparent molecular weight (MW) of RBD-avi-his (heterogeneity ranging from 28 to 38 kDa due to glycosylation) and RBD-Fc (~100 kDa without DTT and ~50 kDa with DTT) are consistent with their theoretically calculated MWs. **(C)** ELISA measurement of binding of the recombinant RBD-avi-his to hACE2-mFc (mouse Fc, Sino Biologicals). 200 ng RBD-avi-his was coated on plate with incubation of serially diluted hACE2-mFc. Binding was detected by using HRP conjugated anti-mouse Fc. Experiments were performed in

duplicate and the error bars denote ± SD, n =2. **(D)** Evaluation of RBD-his and RBD-Fc by size exclusion chromatography. Size exclusion was performed by the Superdex 200 increase 10/300 GL column. The arrows indicate the peaks of the MW standards in PBS. The well-dispersed single peak indicated RBD-his and RBD-Fc exist as monomers in PBS solution.

Figure S2

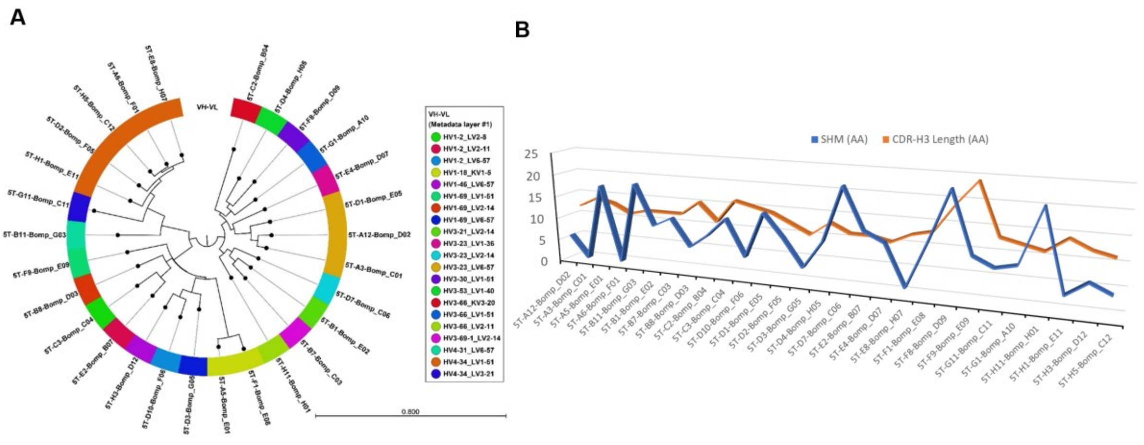

Figure S2. Analysis of the HC/LC usage, and somatic hypermutation (SHM) and CDR-H3 length of 28 SARS-CoV-2 binders selected from our scFv phage library. (A) An unrooted circular phylogram tree was constructed using the VH-VL concatenated sequences of 28 unique anti-SARS CoV-2 RBD scFv clones that were mapped, and color coded by IGHV/IGLV germline paring. **(B)** A 3-D line chart showing the number of SHM and CDR-H3 length in amino acid (AA) for each clone.

Figure S3

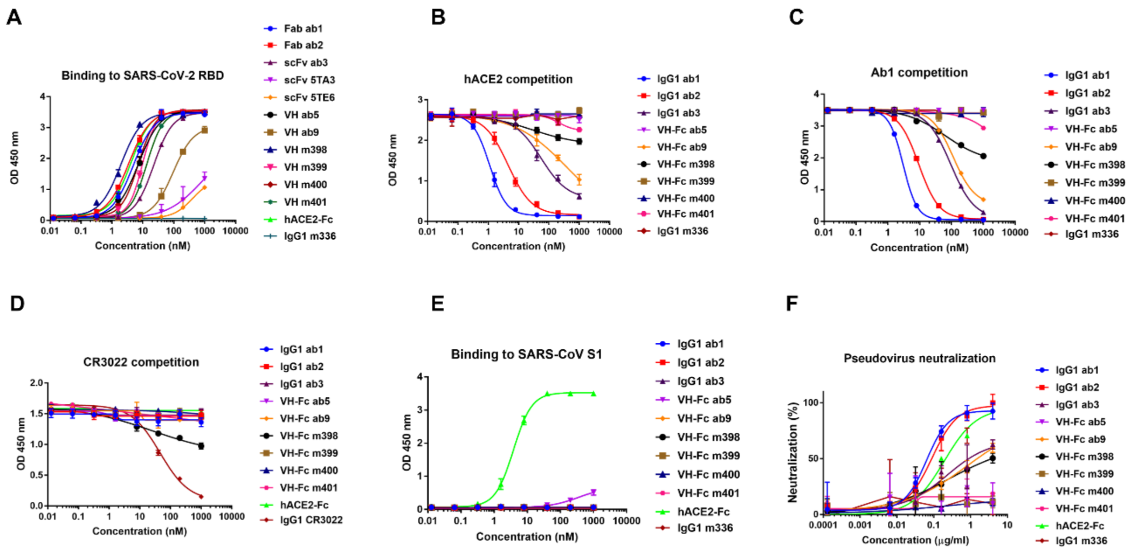

Figure S3. Characterization of a panel of antibodies identified by panning of Fab, scFv, and VH libraries against the SARS-CoV-2 RBD. (A) Binding to SARS-CoV-2 RBD measured by ELISA. 200 ng RBD-Fc was coated and binding was detected by HRP conjugated anti-FLAG antibody. **(B)** Competition of antibodies in IgG1 and VH-Fc formats with hACE2 for binding to RBD measured by ELISA. 200 ng RBD-Fc was coated and antibodies with gradient concentrations in the presence of 2 nM hACE2-mouse Fc were added. Binding was detected by HRP conjugated anti-mouse IgG (Fc specific) antibody. (**C**) Antibodies competition with Fab ab1 for binding to RBD. 200 ng RBD-Fc was coated and antibodies with different concentrations in the presence of ~20 nM Fab ab1 were added. Binding was detected by HRP conjugated anti-FLAG antibody. (**D**) Antibodies competition with Fab CR3022 for binding to RBD. 200 ng RBD-Fc was coated and antibodies of diffeent concentrations in the presence of 10 nM Fab CR3022 were added. Binding was detected by HRP conjugated anti-FLAG antibody. (**E**) Evaluation of

cross-reactivity to SARS-CoV. SARS-CoV S1 was coated and incubated with antibodies IgG1 or VH-Fc or hACE2-human Fc. Binding was detected by HRP conjugated anti-human IgG (Fc specific). In all ELISA experiments, the MERS-CoV antibody IgG1 m336 was used as a negative control. Experiments were performed in duplicate and the error bars denote ± SD, n =2. (**F**) Peudovirus neutralization assays. Pseudoviruses were pre-incubated with antibodies or hACE2-human Fc and then used to infect 293T-ACE2 cells. Infectivity was monitored by the luciferase activities in cell lysates. Experiments were performed in duplicate and the error bars denote ± SD, n =2.

Figure S4

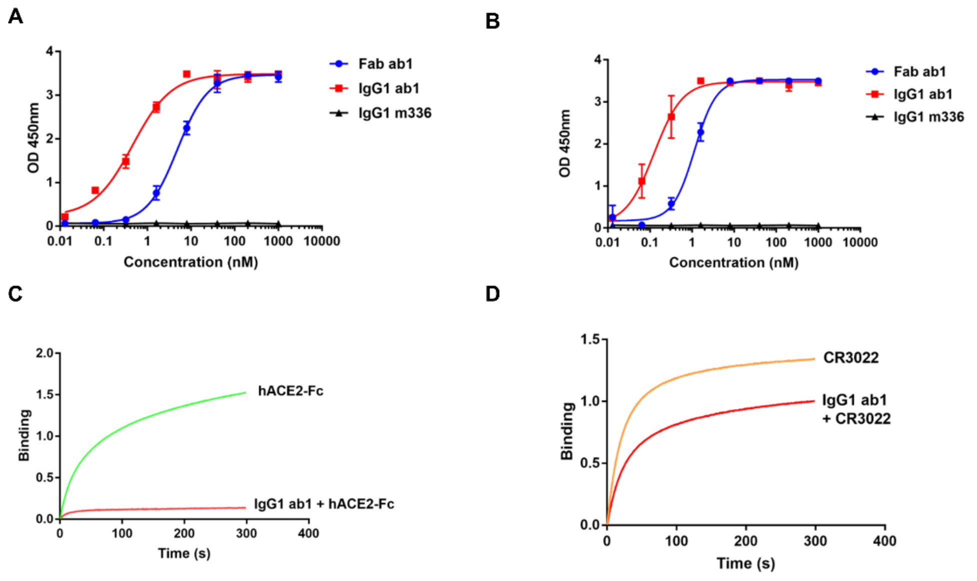

Figure S4. Ab1 binding to SARS-CoV-2 RBD and S1 by ELISA, and measurement of competition with hACE2 and CR3022 by Blitz. (A). Fab and IgG1 ab1 binding to recombinant RBD measured by ELISA. **(B)** Fab and IgG1 ab1 binding to recombinant S1 measured by ELISA. The MERS-CoV antibody IgG1 m336 was used as a negative control. Experiments were performed in duplicate and the error bars denote ± SD, n =2. **(C)** Competition of ab1 with hACE2 tested by Blitz. 100 nM hACE2-Fc was monitored to bind ab1 saturated sensors (red line), which is compared to its independent binding signal to RBD sensor in the absence of ab1(green line). **(D)** Competition of ab1 with CR3022 tested by Blitz. 100 nM Fab CR3022 was monitored to bind ab1 saturated sensors (red line). The signal was compared to the same concentration of CR3022 binding to the RBD sensor in

the absence of ab1 (yellow line). The percentage of signal for CR3022 + ab1 to that of CR3022 alone is ~77%. Thus, there is no competition between CR3022 and ab1.

Figure S5

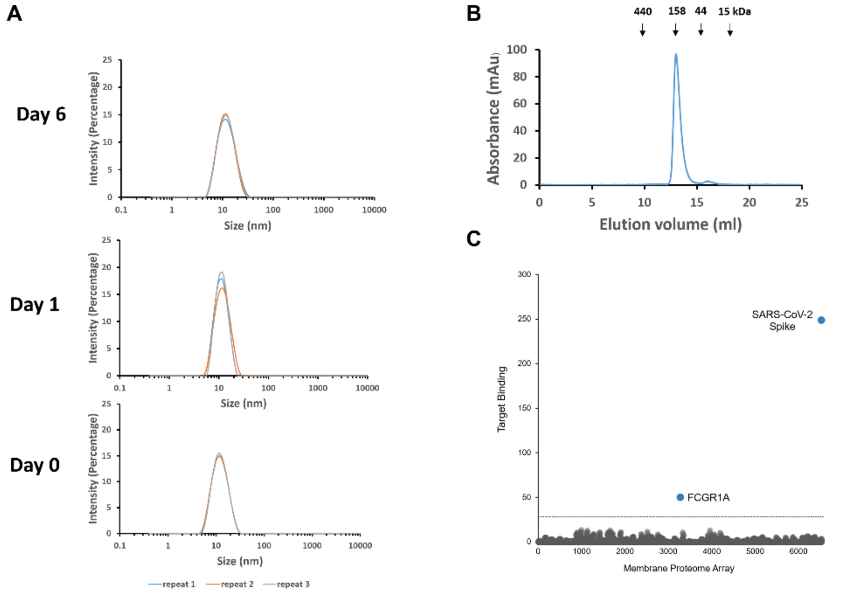

Figure S5. Absent IgG1 ab1 aggregation evaluated by dynamic light scattering (DLS) and size exclusion chromatography (SEC), and lack of ab1 non-specific binding evaluated by a membrane proteome array. (A) Evaluation of the aggregation of IgG1 ab1 by DLS. IgG1 ab1 (2 mg/ml) buffered in PBS was incubated at 37°C. On day 0, day 1 and day 6, samples were taken out for DLS measurement to determine the size distribution. All measurements were repeated three times. **(B)** Evaluation of of IgG1 ab1 aggregation by SEC. Size exclusion was performed by loading proteins (150 µl, 1.5 mg/ml) onto the Superdex 200 increase 10/300 GL column. The arrows indicate the peaks of the MW standards in PBS. **(C)** Lack of non-specific binding measured by a membrane proteome array. Specificity testing of IgG1 ab1 (20 µg/ml) was performed using the Membrane Proteome Array (MPA) platform which comprises 5,300 different human membrane

proteins, each overexpressed in live cells. To ensure data validity, each array plate contained positive (Fc-binding, FCGR1A; IgG1 ab1 binding, SARS-CoV-2) and negative (empty vector) controls. Identified targets were confirmed in a second flow cytometric experiment by using serial dilutions of the test antibody. The identity of each target was also confirmed by sequencing.

SI References

1. Tian X, *et al.* (2020) Potent binding of 2019 novel coronavirus spike protein by a SARS coronavirus-specific human monoclonal antibody. *Emerg Microbes Infect* 9(1):382-385.
2. Ying T, *et al.* (2014) Exceptionally potent neutralization of Middle East respiratory syndrome coronavirus by human monoclonal antibodies. *J Virol* 88(14):7796-7805.
3. Zhang MY, *et al.* (2003) Broadly cross-reactive HIV neutralizing human monoclonal antibody Fab selected by sequential antigen panning of a phage display library. *J Immunol Methods* 283(1-2):17-25.
4. Wu Y, *et al.* (2020) Identification of Human Single-Domain Antibodies against SARS-CoV-2. *Cell host & microbe*.
5. Zhao G, *et al.* (2013) A safe and convenient pseudovirus-based inhibition assay to detect neutralizing antibodies and screen for viral entry inhibitors against the novel human coronavirus MERS-CoV. *Virol J* 10:266.
6. Agrawal AS, *et al.* (2016) Passive Transfer of A Germline-like Neutralizing Human Monoclonal Antibody Protects Transgenic Mice Against Lethal Middle East Respiratory Syndrome Coronavirus Infection. *Sci Rep* 6:31629.
7. Du L, *et al.* (2014) A conformation-dependent neutralizing monoclonal antibody specifically targeting receptor-binding domain in Middle East respiratory syndrome coronavirus spike protein. *J Virol* 88(12):7045-7053.
8. Agrawal AS, *et al.* (2016) Immunization with inactivated Middle East Respiratory Syndrome coronavirus vaccine leads to lung immunopathology on challenge with live virus. *Hum Vaccin Immunother* 12(9):2351-2356.
9. Du L, *et al.* (2013) A Truncated Receptor-Binding Domain of MERS-CoV Spike Protein Potently Inhibits MERS-CoV Infection and Induces Strong Neutralizing Antibody Responses: Implication for Developing Therapeutics and Vaccines. *PLOS ONE* 8(12):e81587.
10. Scobey T, *et al.* (2013) Reverse genetics with a full-length infectious cDNA of the Middle East respiratory syndrome coronavirus. *Proc Natl Acad Sci U S A* 110(40):16157-16162.

11. Yount B*, et al.* (2003) Reverse genetics with a full-length infectious cDNA of severe acute respiratory syndrome coronavirus. *Proc Natl Acad Sci U S A* 100(22):12995-13000.
12. Dinnon KH*, et al.* (2020) A mouse-adapted SARS-CoV-2 model for the evaluation of COVID-19 medical countermeasures. *BioRxiv*:2020. 2005. 2006. 081497.
13. Brochet X, Lefranc MP, & Giudicelli V (2008) IMGT/V-QUEST: the highly customized and integrated system for IG and TR standardized V-J and V-D-J sequence analysis. *Nucleic Acids Res* 36(Web Server issue):W503-508.
14. Raybould MIJ*, et al.* (2019) Five computational developability guidelines for therapeutic antibody profiling. *Proc Natl Acad Sci U S A* 116(10):4025-4030.
15. Tucker DF*, et al.* (2018) Isolation of state-dependent monoclonal antibodies against the 12-transmembrane domain glucose transporter 4 using virus-like particles. *Proc Natl Acad Sci U S A* 115(22):E4990-e4999.
